# Effects of cassava wax bath as a new therapeutic approach on patients with plantar fasciitis: a double-blind, randomised clinical trial

**DOI:** 10.1038/s41598-024-62999-9

**Published:** 2024-05-27

**Authors:** Vitsarut Buttagat, Sittiporn Punyanitya, Rawiwan Charoensup, Supapon Kaewsanmung, Pattanasin Areeudomwong, Sujittra Kluayhomthong

**Affiliations:** 1 Department of Physical Therapy, School of Integrative Medicine, Mae Fah Luang University, Chiang Rai, 57100 Thailand; 2 School of Medicine, Mae Fah Luang University, Chiang Rai, 57100 Thailand; 3 Department of Applied Thai Traditional Medicine, School of Integrative Medicine, Mae Fah Luang University, Chiang Rai, 57100 Thailand; 4 Medicinal Plant Innovation Center of Mae Fah, Luang University, Chiang Rai, 57100 Thailand

**Keywords:** Health care, Medical research

## Abstract

The aim of this study was to investigate the efficacy of a new therapeutic approach (cassava wax bath: CWB) compared with usual care (paraffin wax bath: PWB) in patients with plantar fasciitis (PF). Forty patients with PF were recruited into the study (CWB group, n = 20, PWB group, n = 20). Patients in the CWB group received cassava wax bath and patients in the PWB group received usual care (PWB). The primary outcome was pain intensity (PI). The secondary outcomes were the pressure pain threshold (PPT), pain frequency (PFr), foot and ankle ability measure (FAAM), and ankle dorsiflexion range of motion (ADROM). All outcomes were assessed before and after the five-week intervention, one month, and three months after the intervention period. After the intervention, statistically significant improvement was found in all outcomes after the intervention period and during the one month and three months follow-up study in both groups (P < 0.05). For all outcomes, no between-group differences were seen at any post-assessment time-point, except for PFr (P < 0.05). In conclusion, the findings of this study indicate that CWB was significantly superior to PWB in reducing PFr. For the other outcomes, CWB and PWB were both equally effective in reducing PI and increasing PPT, FAAM, and ADROM in patients with PF. Therefore, CWB might be considered as a novel useful therapeutic option for PF patients.

Trial registration: Thai Clinical Trials Registry (TCTR) (Identification number: TCTR20220128002), First posted date: 28/01/2022.

## Introduction

Plantar fasciitis (PF) is one of the most common causes of heel pain and is characterised by pain at the calcaneus or heel bone resulting from degenerative irritation of the plantar fascia at its origin on the calcaneus^1,2^. The prevalence of PF in the general population has been estimated at 10%^3–5^ This condition is most common in people aged 40–60 years and is slightly more prevalent in women than men^5–8^. The main symptom of PF is under-surface heel pain, which can mostly be seen in the first steps taken in the morning or after a long period of inactivity. After taking a few steps, the pain typically improves. However, the pain can quickly return if prolonged weight-bearing activity is performed^1^. Furthermore, such pain can interfere with a patient’s quality of life^8^. Although the pathogenesis of PF has still not been fully defined^9^ several risk factors, such as improper footwear, obesity, increased age, intrinsic muscle weakness, reduced ankle dorsiflexion, increased foot pronation or supination, reduced first metatarsophalangeal joint extension, and prolonged weight-bearing, are thought to influence the development of PF^10–13^. The treatment options for the management of PF can be divided into the following two main categories: conservative (pharmacologic and non-pharmacologic) and operative treatments. However, Asheghana et al.^9^ and Yildiz et al.^13^ have reported that conservative treatment is generally recommended in the initial treatment of PF and has been advised as the first choice for treatment. In addition, Wongsiri^14^ has revealed that the primary interventions of PF with high success rates in good compliance patients are conservative treatments. These conservative treatments include corticosteroid injections, non-steroidal anti-inflammatory drugs, orthotics, shoe inserts, footwear modification, acupuncture, stretching exercise, strengthening exercises, soft tissue massage, taping, extracorporeal shock wave therapy, low-level laser therapy, cold application, and heat application^2,8,9,13,15^. The application of heat, such as through local warm water baths and paraffin baths, is recommended as the first line of the PF treatment in order to increase tissue flexibility and elasticity and increase blood circulation to promote the healing process^14^.

A paraffin wax bath (PWB) is a kind of superficial heating modality that has been used to treat various musculoskeletal problems such as PF^16–18^. This wax is made from a solid hydrocarbon derived from petroleum^19^. The most common segments to be treated with PWB are the hands and feet^20^. Mir-Bonafe et al.^19^ have reported that PWB can improve local circulation, relieve pain, and promote relaxation. Additionally, Karpuz and Akkurt^21^ evaluated the effects of combined PWB and home exercise on PF patients and found that it reduced pain and increased quality of life more effectively than did home exercise alone.

In Thailand, cassava (*Manihot esculenta* Crantz) has for decades been considered an important economic crop. In 2020, Thailand was the world’s third-largest cassava producer (29 million tonnes per year)^22,23^, and the country has been ranked first in cassava exports, accounting for 57% of the global market share, due to a decline in domestic demand^24^. To add value to cassava exports, in 2022, a new therapeutic heating modality—cassava wax bath (CWB)—with a relatively inexpensive application was first developed. Punyanitya et al^25,26^. have shown that a CWB (glyceryl stearate/cassava starch composite) has a melting point of 51.9 ± 0.3 °C, a latent heat of solidification of 126 ± 3 J/g, and a sustained hot temperature of 9.4 ± 0.5 min (without plastic and towel wrap). Moreover, these authors found that the CWB did not cause any adverse effects to the healthy participants^25^. Nevertheless, it remains unknown whether CWB is an effective treatment for patients with different musculoskeletal disorders including PF. The purpose of the present study was to evaluate the efficacy of CWB compared with PWB in terms of pain intensity (PI), pain frequency (PFr), pressure pain threshold (PPT), foot and ankle ability measure (FAAM), and ankle dorsiflexion range of motion (ADROM) in patients with chronic PF. Based on the preliminary outcomes of our pilot study comparing the effects of CWB with those of PWB in groups of PF patients, we hypothesised that CWB would lead to greater improvement in PI, PFr, PPI, FAAM, and ADROM compared to PWB.

## Methods

### Clinical trial design and setting

A double-blind randomised clinical trial with parallel groups was designed and was carried out between February and December 2022 in the community halls of the Nang Lae sub-districts in Chiang Rai, Thailand. The study was conducted after obtaining approval from the Ethics Committee for Human Research at Mae Fah Luang University (Code: EC 21143-25). The current study was conducted according to the Declaration of Helsinki. Written informed consent was obtained from the study patients.

### Participants

Patients diagnosed with PF were recruited from the Nang Lae Tambon Health Promoting Hospital. The inclusion criteria were as follows: aged between 40 and 70 years, pain upon first rising in the morning in the plantar aspects of the heel for a duration of at least three months, heel pain exacerbated by palpation of the plantar fascia attachment to the medial border of the calcaneus, and willing to participate in the study. The exclusion criteria were as follows: a history of neurological conditions, a history of rheumatic disease, a history of inflammatory arthritis of the lower limb(s), contagious skin disease in the ankle and/or foot, fracture of a lower limb with or without metal implant fixation, inflammation of a lower limb, trauma of the foot or foot surgery, sensory impairment of the ankle and/or foot, and having received any form of treatment (e.g., manual therapy, physical therapy, or medications) in the previous four weeks. 

A power sample calculation was based on the pilot study, which compared the effects of CWB (n = 8) with those of PWB (n = 8) in patients with chronic PF. A standard deviation (of pain intensity) of 2.2 cm for the CWB group and of 1.8 cm for the PWB group was observed. These variances were used to determine the sample size needed to detect a 1.92 cm change in pain intensity. For each group, 20 patients were required; there was a drop-out rate of 15%; 80% power; and a significance level of 0.05.

### Randomisation and blinding

All eligible patients were randomised 1:1 to receive CWB or PWB using block randomisation with block sizes of two and four. Each patient was assigned a number in an opaque and sealed envelope. Randomisation was performed by a research assistant who was not involved in the recruitment, outcome assessments, or treatment processes.

### Intervention

**Figure 1:**
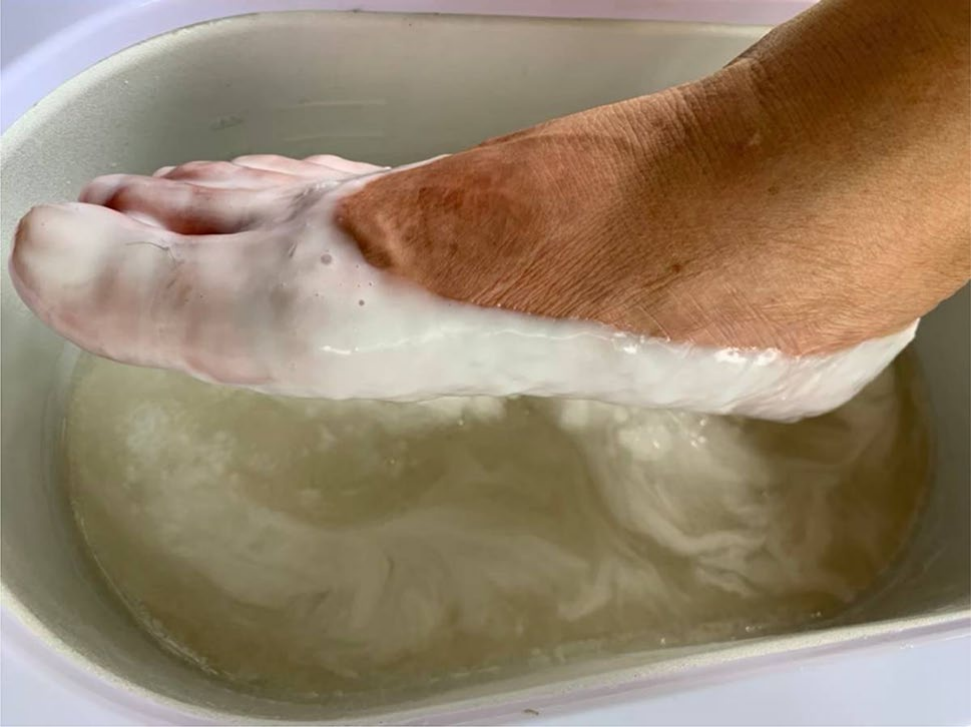
Cassava wax bath.

Patients (n = 20) in the CWB group received CWB (dip and wrap method) three times per week for five consecutive weeks for a total of 15 sessions. The temperature of the CWB was 52 °C. Patients were asked to wash their foot with soap and water and pat their foot dry with a clean towel. Then, patients were asked to submerge the whole foot into the cassava wax for three seconds, withdraw the foot and allow the cassava wax to set (Fig. 1). This procedure was repeated eight times. After the last dip, the foot was wrapped and sealed in plastic bags and covered with a towel for 15 min. For the cassava wax removal, the patients were asked to peel the cassava wax off by themselves. The patients were asked to wash their foot with soap and water after each study visit was completed. The cassava wax was discarded after a single use.

The composite materials prepared and used to produce the cassava wax included cassava starch powder (CSP), glycerin, palm oil, stearic acid, sodium hydroxide (NaOH), and sodium lauryl sulphate. The procedure for the preparation of composites (cassava wax) used in this study has been previously described in more detail by Punyanitya et al.^25,26^ Briefly, first, NaOH was dissolved in distilled water in a beaker; palm oil was then added into the first beaker and mixed at 130 °C. Second, stearic acid and sodium lauryl sulphate were added into the mixture of glycerol and distilled water at 150 °C. Third, the resulting solution was added into the beaker containing NaOH and palm oil and was stirred at 80 °C. The resulting solution was neutralised with citric acid. Fourth, 1% by weight of CSP was dispersed in distilled water. Then, suspended CSP was added into the neutralised solution and stirred at 70 °C. Finally, the mixtures were cooled to room temperature in a mould.

**Figure 2:**
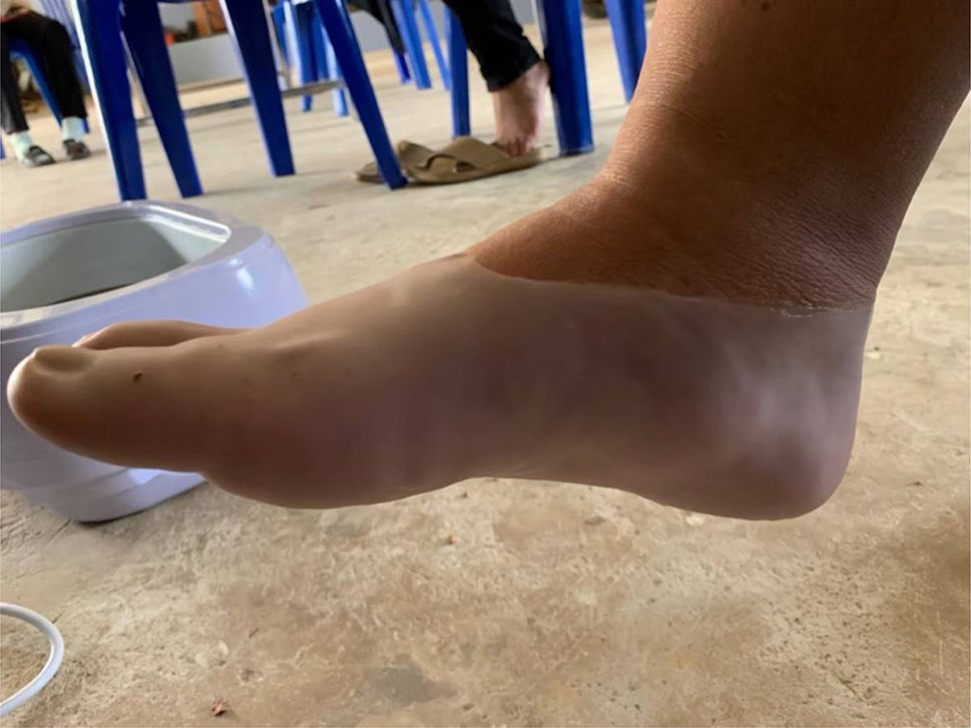
Paraffin wax bath.

Patients (n = 20) in the PWB group received usual care (PWB). Treatment was performed with paraffin wax (Fig. 2) three times a week for five consecutive weeks. The treatment protocol for the PWB group was the same as that for the CWB group. None of the patients had ever been treated with PWB, and the characteristics of the cassava wax and paraffin wax were similar; thus, patients in both groups were unaware of the experimental condition in which they were participating. Moreover, all patients were asked not to participate in any other treatment programmes for the duration of the study.

### Outcome measures

Five measures—PI, PFr, PPT, FAAM, and ADROM—of the treatment effects were assessed at baseline, the end of the treatment program (week 5), one month after the end of the treatment program (one-month follow-up: week 9), and three months after the end of the treatment program (three-month follow-up: week 17). These outcome measures were performed by a research assistant who was blinded to group allocation.

The pain visual analogue scale (VAS) was used to evaluate the PI of the patients. It consists of a 10 cm line with descriptive anchors at each end representing 0 (no pain) and 10 (worst possible pain). The patients were asked to rate their current pain level by placing a mark on the line. The validity and reliability of the data obtained with the pain VAS was shown to be good^27^.

The pain frequency (PFr) was recorded in a daily heel pain diary and was calculated as number per day for each patient during the time frame measured.

The pressure pain threshold (PPT) was measured, using a pressure algometer following the protocol previously described in the literature^28^, at the most painful point in the medial plantar area of the patient’s heel on the affected side or on the foot with the most pain (in cases where the patient had pain on both sides). Each point was assessed two times, and the average was used for analysis.

The Thai version of the Foot and Ankle Ability Measure (FAAM) was used to measure the functional limitations of the patients with chronic PF. The FAAM contains two separate subscales—the activities of daily life (ADL) and the sport subscales. However, in this study, only the ADL subscale was used due to most of the patients in the study being older people and not having played any sport for a considerable length of time. The ADL subscale consists of 21 items ranked on a 5-point Likert scale, evaluating the ability of patients to carry out basic functional activities. A lower score indicates a lower functional level. The Thai version of the FAAM is a reliable and valid tool for identifying changes in functional limitations^29^.

Pain-free passive ankle dorsiflexion range of motion (ADROM) was assessed using the standard goniometer and was obtained with the patient in a long-sitting position with the knee fully extended. This measurement has high reliability (ICC = 0.962)^30^.

Moreover, after the end of the treatment program (week 5), all patients were asked to report any suspected adverse events (AEs) or unexpected events that they experienced during the treatment period.

### Statistical analysis

Data were analysed using the SPSS 20.0 software package (version 20.0; IBM, New York, US) with an alpha level of 0.05. Partial eta-squared values (ηp^2^) were used as measures of effect size and magnitude, interpreted as trivial (< 0.01), small (0.01–0.06), moderate (> 0.06–0.14), and large (> 0.14)^31^.

All data were tested for normality with the Shapiro–Wilk test. Descriptive data are displayed as mean ± standard deviation. To assess baseline group differences, an independent samples t-test was used for the continuous variables, and chi-square tests were used for the categorical variables. Comparisons of quantitative data within the groups were performed using a repeated measures ANOVA with Bonferroni post-hoc analysis. Comparisons of quantitative variables between the groups were performed with an analysis of covariance (ANCOVA) using baseline values as co-variates.

## Results

### Baseline characteristics

**Figure 3:**
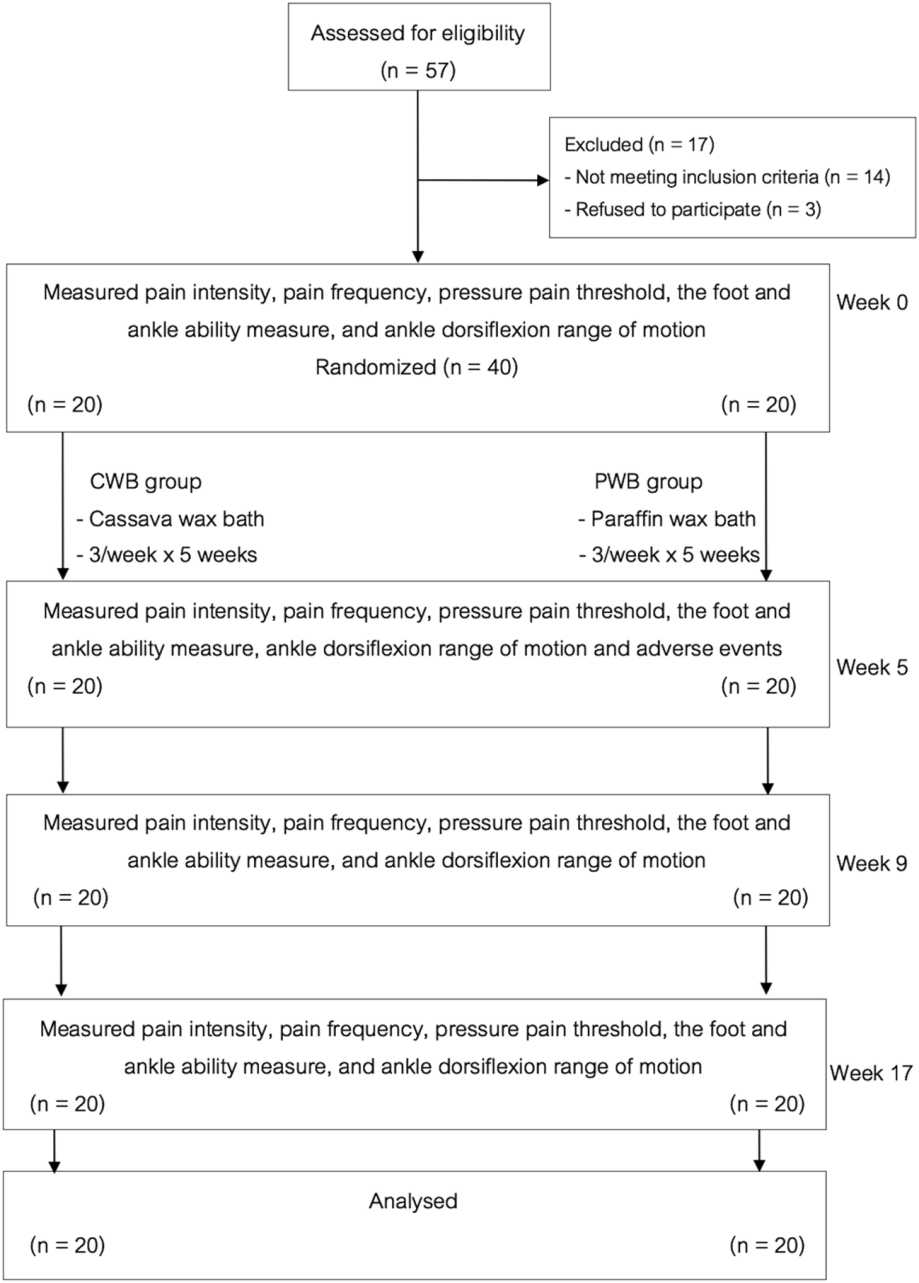
The CONSORT flow diagram.

Fifty-seven patients were assessed for eligibility; 40 were included in the study and were randomly assigned to the CWB (n = 20) or PWB (n = 20) group. During the study period, no patient withdrew from the study. Therefore, all patients (n = 40) were included in the final analysis. There were no side effects observed or reported throughout the study. The relevant CONSORT flow diagram is presented in Fig. 3. No significant differences were found between the groups regarding the baseline data of patient demographics and characteristics (Table 1).

**Table 1:** The baseline data of participant demographics and characteristics (Independent t-tests for continuous data, and chi-square tests for categorical data).

Characteristic	Randomised (n = 40)		P
	CWB (n = 20)	PWB (n = 20)	
Age (years), Mean (SD)	63.80 (3.38)	64.80 (4.34)	0.421
Gender; number of female (%)	14 (70.00)	15 (75.00)	0.091
Weight (kg), Mean (SD)	59.95 (9.73)	63.20 (8.79)	0.275
Height (cm), Mean (SD)	154.55 (6.89)	155.70 (7.06)	0.605
BMI (kg/m^2^), Mean (SD)	25.09 (3.21)	26.06 (3.20)	0.348
Duration of the heel pain episodes (month), Mean (SD)	19.15 ± 14.15	18.95 ± 8.41	0.957

*CWB* Cassava wax bath, *PWB* Paraffin wax bath, *BMI* Body mass index, *kg* Kilogram, *cm* Centimeter, *kg/m*^*2*^ Kilogram per square meter.

### Effects of CWB and PWB

Considering the within-group effects, the PI, PFr, PPT, ADROM, and FAAM all showed significant improvements with treatment in patients in both groups after the end of the intervention period (week 5) and at one month (week 9) and three months (week 17) after the end of the intervention period (p < 0.05) **(**Table 2). For the between-group effects, after adjustment for baseline data, the CWB group showed a statistically significantly greater reduction in PFr than the PWB group at week 5 (p = 0.00; ηp^2^ =0.433), week 9 (p = 0.00; ηp^2^ =0.322), and week 17 (p = 0.00; ηp^2^ =0.318). For the other outcomes, there was no statistically significant between-group difference in terms of the PI, PPT, ADROM, and FAAM at all assessment time points (Table 3).

**Table 2:** Comparison of the outcome measures between baseline (pre-test) and post-test assessments in the CWB and PWB groups (Repeated Measures ANOVA).

Outcome measures	Groups	Baseline	Week 5 (Post-test 1)	Week 9 (Post-test 2)	Week 17 (Post-test 3)
Pain intensity (VAS): Mean ± SD	CWB	5.90 ± 1.65	0.80 ± 1.28*	1.05 ± 1.79*	1.35 ± 2.01*
	PB	5.95 ± 1.82	1.10 ± 1.59*	0.80 ± 1.20*	1.05 ± 1.64*
Pain frequency (PFr): Mean ± SD	CWB	6.85 ± 2.08	0.90 ± 0.97*	1.55 ± 0.94*	1.70 ± 1.13*
	PB	6.55 ± 2.04	2.95 ± 1.67*	3.15 ± 1.42*	3.30 ± 1.38*
Pressure pain threshold (PPT): Mean ± SD	CWB	4.77 ± 2.33	8.48 ± 2.18*	8.26 ± 2.62*	9.21 ± 2.81*
	PB	5.87 ± 3.29	8.81 ± 2.75*	8.24 ± 2.31*	9.03 ± 3.69*
Ankle dorsiflexion (ADROM): Mean ± SD	CWB	11.86 ± 8.23	19.41 ± 4.75*	18.48 ± 4.18*	17.93 ± 5.88*
	PB	12.46 ± 9.82	19.236.22*	18.40 ± 6.57*	17.51 ± 6.60*
The Foot and Ankle Ability Measure (FAAM): Mean ± SD	CWB	50.75 ± 16.90	78.20 ± 5.57*	79.20 ± 6.21*	81.60 ± 3.69*
	PB	46.90 ± 20.69	78.45 ± 5.59*	80.95 ± 3.75*	82.30 ± 3.11*

CWB Cassava wax bath, PWB Paraffin wax bath.

*Significant improvement from baseline levels (P < 0.05).

**Table 3:** Comparison of mean post-test measures at each assessment time point between the CWB (n = 20) and PWB (n = 20) groups after adjustment for differences in baseline values (ANCOVA).

Outcome	Week 5 (Post-test 1)			Week 9 (Post-test 2)			Week 17 (Post-test 3)		
	CWB^#^ (Mean ± SD)	PWB^#^ (Mean ± SD)	Difference (95%CI; ηp^2^)	CWB^#^ (Mean ± SD)	PWB^#^ (Mean ± SD)	Difference (95%CI; ηp^2^)	CWB^#^ (Mean ± SD)	PWB^#^ (Mean ± SD)	Difference (95%CI; ηp^2^)
Pain intensity (VAS)	0.81 (1.42)	1.10 (1.42)	-0.29(-1.20–0.62; 0.011)	1.10 (1.51)	0.80 (1.51)	0.30(-0.78–1.23; 0.008)	1.35 (1.85)	1.05 (1.85)	0.30(− 0.88–1.49; 0.007)
Pain frequency (PFr)	0.86 (1.27)	2.99 (1.27)	-2.13*(-2.94–-1.32; 0.433)	1.54 (1.22)	3.16 (1.22)	- 1.62*(-2.40–-0.84; 0.322)	1.68 (1.25)	3.32 (1.25)	− 1.64* (2.44–− 0.84; 0.318)
Pressure pain threshold (PPT)	8.59 (2.46)	8.68 (2.46)	-0.09(-1.68–1.50; 0.000)	8.40 (2.67)	8.10 (2.67)	0.30(-1.26–1.86; 0.004)	9.46 (3.08)	8.77 (3.08)	0.69(− 1.30–2.68; 0.013)
Ankle dorsiflexion (ADROM)	19.55 (3.50)	19.08 (3.50)	0.47(-1.77 – 2.71; 0.005)	18.59 (4.41)	18.28 (4.41)	0.31(-2.52–3.14; 0.001)	18.05 (5.12)	17.39 (5.12)	0.66(− 2.63–3.95; 0.004)
The Foot and Ankle Ability Measure (FAAM)	78.10 (5.58)	78.54 (5.58)	-0.44(-4.03–3.15; 0.002)	79.13 (5.17)	81.01 (5.17)	-1.88(-5.21–1.43; 0.035**)**	81.57 (3.46)	82.32 (3.46)	− 0.75(2.97–1.48; 0.012)

*CWB* Cassava wax bath, *PWB* Paraffin wax bath, ηp^2^ Partial eta-squared values.

^#^CWB and PWB data are the mean post-test values after adjustment for differences in baseline values (adjusted post-test values) by ANCOVA. Therefore, SDs of these mean post-test measures provided from ANCOVA analysis are equal across the two groups.

*Significant difference between groups (P < 0.05).

## Discussion

This study investigated the effects of CWB on PI, PFr, PPT, ADROM, and FAAM compared to the effects of usual care among patients with chronic PF. The results revealed that the application of CWB, the study’s proposed new therapeutic option, for five weeks provided an improvement in PI, PFr, PPT, ADROM, and FAAM scores in this patient population, and the improvement was maintained for at least three months. The findings of the current study provide the first evidence that CWB as studied here can have therapeutic effects for chronic PF patients. The results of the study support the treatment guidelines of Wongsiri^14^, who postulated that heat therapy, such as PWB or hot water bath, should be considered as the first-line treatment option and as an effective intervention for PF patients.

As yet, no similar studies have been reported. In particular, no research is available in the literature evaluating the effects of this new therapeutic heating modality—CWB—for the treatment of PF; thus the findings of this study cannot be directly compared to the results of other studies. However, as PWB and CWB are the same kind of therapeutic heating modalities and the general characteristics of these two modalities are similar, the results of PWB studies were compared. With regard to PWB, the findings of the present study appear to be in line with previous studies. Karahan et al.^32^ have reported that PWB applied five times a week for a duration of three weeks improved pain levels, as well as the results of the Heel Tenderness Index and Foot and Ankle Outcome Score in PF patients. Furthermore, Khatri and Shukla^1^ examined the effects of PWB on patients with PF. They found that PI, PPT, and functional disability were significantly improved in patients with PF treated with 10 PWB sessions. It has been reported in the literature that PWB also has beneficial effects on other populations. Li et al.^33^ investigated the effects of PWB in healthy participants. They revealed that a statistically significant decrease in gastrocnemius muscle belly and Achilles tendon stiffness was found after the participants received PWB. Dilek et al.^17^ reported that the application of PWB had a greater effect in decreasing pain and tenderness and maintaining muscle strength than control group in patients with hand osteoarthritis. Sibtain et al.^20^ carried out a clinical trial to investigate the effects of PWB combined with joint mobilisation. They found that PWB combined with joint mobilisation was more effective in reducing pain and increasing wrist range of motion than joint mobilisation alone in patients with post-traumatic stiff hand.

Although the mechanisms behind the improvement of pain, mobility, and function in PF are not well understood^34^, there are some theoretical considerations that might explain the findings of the current study. Wongsiri^14^ and Malanga, Yan, and Stark^35^ have reported that superficial heating provided by hot baths can stimulate reflex vasodilation and thus produce greater blood flow and heat throughout the area to be treated, which is believed to accelerate healing by increasing the supply of oxygen and nutrients to the affected site. The local tissue metabolism rate can also be increased by heating, which might promote healing^35^. Additionally, Dilek et al.^17^ and Li et al.^33^ have stated that heat application, such as PWB, could induce muscle relaxation due to the vasodilatation of the peripheral blood vessels. This may be a factor leading to improvement in parameters evaluated in the present study. Another potential mechanism is that pain may be reduced by the washing away of pain-producing substances with improved blood circulation.^36^ Concerning the feasible mechanisms underlying the effects of CWB and PWB on ADROM, Bleakley and Costello^37^ and Hardy and Woodall^38^ have postulated that an improvement in range of motion after heat therapy may be associated with changes in the viscoelastic properties of collagenous tissues.

Considering the between-group effects, CWB was significantly better for those with PF in terms of PFr reduction in comparison with PWB following treatment, suggesting that CWB was superior to PWB regarding PFr. Iswarya et al.^39^ have reported that during the solidification of the wax, the energy of the latent heat of the wax is released, and its heat energy is conducted into the patient’s tissues. Punyanitya et al.^26^ have revealed that, according to a differential scanning calorimeter analysis, the cassava wax used in the present study had a higher latent heat of solidification (126 J/g) than paraffin wax (121 J/g), suggesting that the heat energy being conducted into the patients’ tissues in the CWB group might be higher than in the PWB group. Moreover, based on observation of the patients dipping their feet into the wax, although the number and duration of the dips were equal between the groups, the amount of cassava wax that had congealed and covered the foot of all patients in the CWB group (Fig. 1) was obviously greater than the amount of paraffin wax that had congealed and covered the foot of all patients in the PWB group (Fig. 2). As the amount of latent heat released during the solidification of the wax is associated with the amount of wax congealed on the treatment area^39,40^. Thereby, this implies that the amount of heat energy released and conducted into the patients’ tissues in the CWB group was more than that released and conducted into the patients’ tissues in the PWB group. Gucker^41^ has revealed that the heat penetration and the increase in tissue temperature and local circulation of the treated area depend on the amount of heat applied. Furthermore, Lehmann et al.^42^ have also reported that the most effective therapeutic modality that should be chosen is the one that generates the greatest heat at the treatment area without exceeding tolerance levels. Accordingly, it is possible that the greater heat energy released during CWB compared with that released during PWB may be related to the greater reduction of the PFr in the CWB group compared to the PWB group. For the other outcomes—PI, PPT, ADROM, and FAAM—there was no statistically significant difference between the CWB and PWB groups at any post-assessment time point. We therefore suggest that these two interventions may have similar therapeutic efficacy in terms of these outcomes and could be applied interchangeably as therapeutic heating modalities ﻿for the treatment of chronic PF.

The primary strength of the present study is the randomised clinical trial design with patients and assessor blinded; therefore the findings observed are largely immune to bias. Another strength of this study is that there were no patients lost to follow-up. Therefore, this can improve the robustness of the data obtained from the current study. However, the present study also has some limitations. First, the experiment was not designed to include a real control group. Thus, the extent of the improvement of the patients’ outcomes through natural recovery could not be verified. However, all the study participants were in the chronic phase and had persistent heel pain for at least 11 months without natural recovery. The average duration of the heel pain episodes of the patients in the CWB and PWB groups were 19.15 ± 14.15 and 18.95 ± 8.41, respectively. Thus, the chances that their outcomes would improve during this study through natural recovery were small. Second, the study included solely older chronic PF patients (aged between 40 and 70 years). The results can only be generalised to chronic PF patients with similar ages as those in the present study. Further studies will be needed to include participants with different populations. Third, in this study, we evaluated the long-term outcomes up to only three months, which means that the results may not reflect longer-term consequences of the interventions; furthermore, this period of time (3 months) may not be long enough to see any recurrence of PF symptoms. Thus, a longer follow-up period (e.g., 1 year) should be used in a future study. Lastly, regarding the assessment of AEs in the present study, patients were asked to report AEs after the end of the treatment programme (week 5). Some AEs may have been forgotten by some patients, meaning that the number of reported AEs may not reflect the actual frequency of AEs. Therefore, in a future study, patients should be asked during every study visit about their experience of AEs immediately after the end of each treatment session.

## Conclusion

These results suggest that the novel therapeutic heat modality CWB is a safe method and can have several advantages including reducing pain and pain frequency, increasing PPT and ADROM, and improving the physical function of patients with chronic PF. Therefore, CWB might be considered as a useful therapeutic option for improving PI, PFr, PPT, ADROM, and function in chronic PF patients.

## Data Availability

The dataset used in this study are available from the corresponding author on reasonable request.
